# A State Sanatorium for Consumptives

**Published:** 1907-01

**Authors:** 


### A STATE SANATORIUM FOR CONSUMPTIVES.

Thousands of consumptives of the indigent class flock to Texas every year on the approach of winter, and they are a constant menace to the public health. They infect the cars, boarding houses, parks, public places, and, in fact, everywhere, so that any person is liable at any time, anywhere, to acquire the disease.

It is not so much a humanitarian proposition that they should be cared for, as a measure of protection to the public. The disease is not "catching," like smallpox or measles, but it is infectious, and the infection is in what they cough up or spit out, as well, perhaps, as other dejecta. If you extract the fangs of a serpent you disarm him. If the infectious element,—the sputa, principally, of a consumptive, be destroyed,—he is harmless. It is therefore necessary to the protection of the general public that these circulating foci of infection be "retired," corralled, looked after; not only that they may have a showing for recovery, but that they may not spread the disease.

The whole world is aroused now to the necessity of measures to prevent or limit the spread of tuberculosis. Texas has always taken an active part in the campaign, and it should be a matter of pride with her lawmakers that she shall be one of the first to take active steps in the matter.

When the duty and expense of quarantining against yellow fever is relegated to the Federal government, as it should be, and I believe will be, by the Thirtieth Legislature now in session, the money usually appropriated for quarantine can be used to great advantage in other ways, as pointed out elsewhere in this issue.

The Twenty-ninth Legislature passed an act giving to the Quarantine Department of Texas the revenues from inspection of vessels at Galveston for two years. There is $38,000 of this money now in the State Treasury to the credit of that fund; and easily $12,000 will be turned in before the change takes place. The Legislature will doubtless ratify the transfer of the gulf and border quarantine to the Federal government. The government will buy the stations and equipments. It is suggested and urged that the proceeds of such sale be added to the fund above mentioned, and invested in a State sanatorium for the indigent consumptives: that the State set aside a large tract of its vacant land in the well-known salubrious section of West Texas, say 100,000 acres, and that necessary buildings and equipment be provided; the fund mentioned should be sufficient. Let it be an out-of-doors sanitarium—tents in winter; the canopy of heaven, only, to sleep under in good weather. It is well known that employment (occupation for mind and hands) contributes to the cure of this disease. Let the inmates farm,—manufacture such things as are usually made in our other institutions,—an industrial school could be inaugurated, and thus a revenue could be provided which would make the institution partly self-sustaining. There should be diversion, also amusements: the invalids could hunt, fish, ride "hike o'er the hills," etc. But that is mere detail.

The suggestion is urged, and I hope some influential member of the Legislature will promptly act upon it. The passage of such measure will immortalize the man who introduces and secures it.

Four hundred and eleven people die of consumption in the United States every day: a thousand every two days and a half: one every three minutes, night and day; and yet consumption is a preventable disease! Let Texas do something to stop *this fearful waste* of life, and protect the homes of her people.

